# Amelioration of T lymphocyte subsets, inflammatory factors and gastrointestinal function by traditional Chinese medicine rehabilitation management in postoperative chemotherapy for lung cancer

**DOI:** 10.5937/jomb0-56918

**Published:** 2025-08-21

**Authors:** Xiangni Zou, Yuyang Tang, Pei Shi

**Affiliations:** 1 Heilongjiang University of Chinese Medicine, First Affiliated Hospital, Department of Nursing, Haerbin, Heilongjiang, China; 2 Heilongjiang University of Chinese Medicine, First Affiliated Hospital, Department of Oncology, Haerbin, Heilongjiang, 150040, China

**Keywords:** T lymphocyte subsets, inflammatory factors, oxidative stress, lung cancer, rehabilitation, podskupovi T limfocita, inflamatorni faktori, oksidativni stres, karcinom pluća, rehabilitacija

## Abstract

**Background:**

To analyse the influence of Traditional Chinese Medicine (TCM)-characteristic rehabilitation management on T lymphocyte subsets, inflammatory response, and stress response during the postoperative chemotherapy of lung cancer (LC).

**Methods:**

189 LC patients admitted to our hospital from July 2023 to June 2024 were enrolled in this research. Among them, 107 patients who did not receive TCM-characteristic rehabilitation management were regarded as the control group, while the other 82 patients who underwent such management were designated as the observation group. The levels of inflammatory factors and stress response markers before and after treatment in both groups were compared, including hypersensitive-C reactive protein (hs-CRP), interleukin-6 (IL-6), superoxide dismutase (SOD), malondialdehyde (MDA), and glutathione peroxidase (GSH-Px). The T lymphocyte subsets [CD3+, CD4+, CD8+, and natural killer (NK) cells] and gastrointestinal function were also detected.

**Results:**

After the completion of treatment, it was observed that the levels of hs-CRP MDA, and the proportion of CD8+ T lymphocyte subsets in the observation group were significantly reduced compared with the control group (P< 0.05). In contrast, the activities of SOD and GSH-Px, the proportions of CD3+ and CD4+ T lymphocyte subsets, as well as the activity of NK cells in the observation group were notably higher versus the control group (P< 0.05). Moreover, the gastrointestinal function of the patients in the observation group exhibited a more favourable condition after treatment (P< 0.05).

**Conclusions:**

TCM-characteristic rehabilitation management can effectively ameliorate inflammatory responses and stress injury in patients undergoing postoperative chemotherapy for LC and enhance their immune and gastrointestinal functions.

## Abstract (sr)

**Uvod:**

Cilj ove studije je analiza uticaja rehabilitacionog upravljanja karakterističnog za tradicionalnu kinesku medicinu (TCM) na podskupove T limfocita, inflamatorni odgovor i odgovor na stres tokom postoperativne hemioterapije karcinoma pluća (LC).

**Metode:**

U istraživanje je uključeno 189 pacijenata sa LC koji su primjeni u našu bolnicu u periodu od jula 2023. do juna 2024. godine. Među njima, 107 pacijenata koji nisu bili podvrgnuti rehabilitacionom upravljanju karakterističnom za TCM su činila kontrolnu grupu, dok j'e preostalih 82 pacijenata, koji su prošli ovu vrstu rehabilitacije, činilo posmatranu grupu. Upoređeni su nivoi inflamatornih faktora i markera odgovora na stres pre i nakon tretmana u obe grupe, uklj'učuj'ući visokosenzitivni C-reaktivni protein (hs-CRP), interleukin-6 (IL-6), superoksid dismutazu (SOD), malondialdehid (MDA) i glutation peroksidazu (GSH-Px). Takođe su ispitani podskupovi T limfocita [CD3+, CD4+, CD8+ i prirodne ćelije ubice (N K)], kao i gastrointestinalna funkcija.

**Rezultati:**

Nakon završetka tretmana, u posmatranoj grupi su zabeležena značajna smanjenja nivoa hs-CRP, MDA i procenta CD8+ T limfocita u poređenj'u sa kontrolnom grupom (P< 0,05). Nasuprot tome, aktivnosti SOD i GSHPx, procenti CD3+ i CD4+ T limfocita, kao i aktivnost NK ćelija u posmatranoj grupi su bile značajno više u odnosu na kontrolnu grupu (P< 0,05). Pored toga, gastrointesti - nalna funkcija pacijenata u posmatranoj grupi je pokazala povoljnije stanje nakon tretmana (P< 0,05).

**Zaključak:**

Rehabilitaciono upravljanje karakteristično za TCM može efikasno da ublaži inflamatorne odgovore i oštećenja izazvana stresom kod pacijenata na postoperativnoj hemioterapiji LC, kao i da poboljša njihovu imunološku i gastrointestinalnu funkciju.

## Introduction

Lung cancer (LC) represents one of the most prevalent malignant tumours globally. In China, statistics show that newly diagnosed LC cases exceeded 828,000 in 2024, ranking first in incidence among all malignancies [1]. Meanwhile, LC is a disease associated with an exceedingly high mortality risk, with an average mortality rate of 40 per 100,000 [2]. Currently, the principal therapeutic approach for LC in clinical practice remains the combination of surgical resection, radiotherapy, and chemotherapy. Nevertheless, chemotherapy agents typically exhibit neurotoxic properties. While killing tumour cells, they also damage normal cells, leading to a decrease in patients' immunity and an aggravation of inflammation [3]. These effects not only result in frequent complications such as myelosuppression, gastrointestinal reactions, and hepatic function impairment but also impose a substantial psychological burden on patients, ultimately leading to an unfavourable prognosis [4]. In light of this, it has been proposed in clinical practice that during the postoperative chemotherapy process, targeted intervention measures should be given to patients to eliminate or alleviate the impact of chemotherapy-induced side effects, thereby improving patient prognoses [5].

Traditional Chinese Medicine (TCM)-characteristic rehabilitation management is a kind of TCM management based on the theory of TCM, which implements TCM auxiliary therapies (acupuncture and moxibustion, massage, oral Chinese medicine, etc.) and targeted TCM management strategies for patients, with the advantages of high safety, relatively low cost, and operational simplicity [6]. Many domestic studies have reported the positive outcomes of TCM-characteristic rehabilitation management in clinical applications. For instance, Tao WW et al. [7] have demonstrated that TCM-characteristic rehabilitation management is conducive to enhancing the quality of life of cancer patients. Additionally, Kwok JYY et al. [8] have shown that it can effectively relieve the negative emotions of patients with Parkinson's disease. However, its application in LC has been scarcely documented hitherto. Nonetheless, from previous research efforts, it is evident that TCM-assisted therapies possess remarkable efficacy in ameliorating the side effects of LC chemotherapy and attenuating inflammation [9] [10], which also lays a solid foundation for the utilisation of TCM-characteristic rehabilitation management in the postoperative chemotherapy of LC.

It is well known that inflammatory response, immune function and stress response play a significant role in LC chemotherapy. LC patients are often accompanied by chronic inflammation, and chemotherapeutic drugs may further exacerbate inflammation, leading to increased levels of cytokines, which affects the effect of chemotherapy [11]. The T-lymphocyte subpopulation plays a key role in tumour immunity, and chemotherapeutic drugs may inhibit T-cells' function, weakening the immune system's killing effect on the tumour. Still, they may also enhance the immune response by releasing tumour antigens [12]. The immune system's tumour-killing effect may also enhance the immune response by releasing tumour antigens [13]. For stress response, physiological and psychological stress caused by chemotherapy may activate the hypothalamic-pituitary-adrenal axis (HPA axis), leading to increased cortisol levels and suppression of immune function [14]. At the same time, chemotherapy not only brings about physical stress but may also lead to anxiety and depression, which further affects immune function and treatment efficacy [15]. In other words, in the process of chemotherapy, inflammatory response, immune function, and stress response interact and influence each other, which not only affects the effect of chemotherapy but also is a key factor in determining the prognosis of patients.

In the present study, we will conduct an objective and comprehensive evaluation of the diverse impacts of TCM-characteristic rehabilitation management on the inflammatory response, T lymphocyte subsets, and stress response during the postoperative chemotherapy of LC. These findings will confirm for the first time the value of TCM-characteristic rehabilitation management in LC chemotherapy and give future clinics new management strategies in the treatment of LC, thus optimising the prognosis of LC patients [16] [17].

## Materials and methods

### Research subjects

A retrospective cohort study was carried out in this research, with the research subjects being patients diagnosed with LC who were admitted to our hospital from July 2023 to June 2024. Through calculation using the G*Power software (the test family is a t-test with parameter settings: tail=2, effect size=0.3 (medium size test), α = 0.05, power: 0.85), it was confirmed that a minimum of 134 research subjects were necessary for this study (at least 67 subjects in each group). After being screened following the inclusion and exclusion criteria, 189 LC patients were finally included. Among them, 107 patients who did not receive TCM-characteristic rehabilitation management were designated as the control group, and another 82 patients who underwent such management were identified as the observation group. This study has been approved by our Ethics Committee (2023034-07) and will comply strictly with the Declaration of Helsinki. All study subjects signed an informed consent form.

### Inclusion and exclusion criteria

Inclusion criteria: Patients with LC who were diagnosed by pathological examination and who underwent chemotherapy after surgical operation with an anticipated survival period exceeding six months were enrolled. Exclusion criteria: The following patients were excluded: (1) Those with severe diseases such as serious heart, liver, kidney, and hematopoietic system disorders. (2) Those with other malignant tumors. (3) Those with allergic constitutions or allergies to multiple drugs. (4) Those with eczema, ulcers, chilblains, blisters, etc., on the external ear were unsuitable for auricular point sticking and pressing. (5) Those with local skin ulceration, rashes, oedema, blisters, etc., making them inappropriate for application, massage, and other related therapies.

### Ethical statement

This study has obtained the approval of our hospital's Ethics Committee. Patient-informed consent was not mandatory since this is a retrospective study and the data were derived from pre-existing medical records. All patient data were anonymised to guarantee the complete protection of patient privacy.

### Methods

Patients were administered platinum-based chemotherapy regimens, which included but were not limited to Cisplatin (Jiangsu Hao Sen Pharmaceutical Group Co., Ltd., Batch No.: 601240106), Carboplatin (Qilu Pharmaceutical Co., Ltd., Batch No.: AB1J3022), and Nedaplatin (Nanjing Xian Sheng Dong Yuan Pharmaceutical Group Limited, Batch No.: 193-230303). Each treatment cycle lasted 21 days, and chemotherapy was conducted continuously for 3 to 5 cycles. Control group: Conventional TCM treatment was adopted, which included the following modalities: (1) Acupoint acupuncture: Acupuncture was administered at specific acupoints, namely Jianjia, Jianliao, Jianyu, and Binao. The acupuncture needles were retained for 30 minutes. Subsequently, massage was performed on Ashi, Weishu, Pishu, Neiguan, and Zusanli acupoints, with each massage session lasting for 2-3 minutes. (2) Auricular acupressure therapy: Auricular points associated with the lung, spleen, stomach, Shenmen, and endocrine were selected. After disinfecting these auricular points with 75% alcohol, Vaccariae semen (Jiangxi Zhang Shu Tian Ql Tang TCM Decoction Pieces Co., Ltd., Batch No.: 2211005) was applied to the chosen points and then massaged until a local perception of soreness, numbness, distension, pain, or a burning sensation was elicited. The seeds were replaced every other day. (3) Acupoint application: Processed Radix Aconiti (Zhang Shu Tian Ql Tang TCM Decoction Pieces Co., Ltd., Batch No.: 22091005), Radix Aconiti Kusnezoffii Preparata (Jiangxi Zhang Shu Tian Ql Tang TCM Decoction Pieces Co., Ltd., Batch No.: 22081003), and fresh ginger were pulverised into an appropriate size and then positioned on the Shenque acupoint, where they were retained for 4-6 hours. The acupoint application was initiated 1 hour before the commencement of chemotherapy. (4) Drug guidance and monitoring of signs and complications: Regular monitoring of patient's physical signs was accompanied by professional guidance regarding drug usage and vigilant surveillance for potential complications. Observation group: Based on the control group, the strategies of »informing, conversing, guiding, and enlightening« were employed to interact with patients following the principles expounded in »Huang Di Nei Jing« to help patients face up to the disease, actively cooperate with the treatment, and relieve their psychological pressure. The details are as follows: (1) Environmental management: The ward environment was meticulously maintained to ensure comfort and tranquillity, creating a conducive atmosphere for patient recovery. (2) Psychological management: Active communication and exchanges were conducted with patients and their families, enabling a comprehensive understanding of their negative emotional states. Subsequently, appropriate measures were taken to assist patients in effectively expressing and releasing their inner negative emotions. Additionally, the detrimental impacts of negative emotions on disease treatment were elucidated to patients and their families. This was followed by guiding family members to assume an active role in providing encouragement and solace to patients, thereby promoting a positive attitude among patients towards disease treatment and nursing care. (3) Emotional management: Tailored music therapy regimens were designed based on patients' preferences and disease conditions. Patients were advised to listen to more than five pieces of soothing and melodious music daily. Meanwhile, scientific and empathetic language counselling was provided to assist patients in redirecting their emotions and reducing psychological stress. Patients were inspired to foster a firm resolve to combat the disease through moderate comfort and encouragement. In cases where patients exhibited insomnia symptoms or difficulty falling asleep, they or their family members were instructed to massage the patients' Yongquan acupoint for five minutes before bedtime to facilitate sleep induction. (4) Dietary management: Foods with cooling and fluid-nourishing properties were prescribed. For instance, a porridge prepared by combining Rehmannia juice and japónica rice was recommended to nourish yin and generate fluids. Additionally, family members were asked to prepare a wolfberry and lily porridge as part of the dietary regimen. Astragalus, Chinese dates, and American ginseng could also be utilised for brewing tea, which enhanced the patient's overall health and resilience. (5) Follow-up management: Modules such as health guidance, health management, specialised lectures, online consultations, and medication reminders were integrated into the hospital's online platform or WeChat follow-up group. Patients were systematically trained to utilise these modules proficiently. Subsequently, relevant and up-to-date knowledge regarding LC, chemotherapy, and TCM rehabilitation treatment was pushed to patients via the online platform for their perusal. An online consultation platform was also established to optimise prognostic management and ensure continuous medical support and patient education.

### Sample collection and detection

Patients' fasting venous blood was collected before and after treatment (specifically, after three chemotherapy cycles) and divided into three portions. One portion was used for counting T lymphocyte subsets (CD3^+^, CD4+, CD8^+^, and NK) by flow cytometry (CytoFLEX, Beckman Coulter, USA). Another portion was centrifuged to separate serum, following which gastrin-17 (G17) and pepsinogen l/ll (PGI/II) levels were determined via an automated biochemical analyser (BS-2000M, Myriad, USA). The third portion was used for measuring hypersensitive-C reactive protein (hs-CRP), interleukin-6 (IL-6), superoxide dismutase (SOD), malondialdehyde (MDA), and glutathione peroxidase (GSH-Px) by enzyme-linked immunosorbent assay (ELISA). These assay kits were all purchased from Shanghai Xuanke Biotechnology Co., Ltd, China, and the entire operational procedure strictly followed the accompanying kit instructions.

### Outcome measures

(1) The inflammatory reaction (hs-CRF) TNF-α, and IL-6), stress reaction (SOD, MDA, and GSH-Px), T lymphocyte subsets (CD3^+^, CD4^+^, CD8+, and NK), and gastrointestinal function (G-17, PGI, and PGII) were compared between the two groups before and after treatment. (2) The Self-rating Anxiety Scale (SAS)/Self-rating Depression Scale (SDS) (16) and the Pittsburgh Sleep Quality Index (PSQI) (17) were employed to assess patients' psychological state and sleep quality after treatment. Both the SAS and SDS contain 20 items on a four-point scale. The scores of the 20 items were summed to obtain a total crude score, which was then multiplied by 1.25 and rounded to the nearest whole part to obtain a standardised score. When the standardised score is greater than the cut-off value (SAS: 50, SDS: 53), higher scores indicate more severe anxiety/depression. The PSQI consists of seven dimensions, each scored from 0 to 3, with a total score ranging from 0 to 21. Higher scores indicate poorer sleep quality.

### Statistical methods

This study employed SPSS 25.0 software (IBM, USA) for statistical analysis. The chi-square test was used to compare categorical data presented as [n(%)]. The independent sample t-test and paired t-test were used for the comparison of normally distributed continuous data denoted as (x̄±s). Regarding non-normally distributed continuous data expressed as [median (interquartile range)], the Mann-Whitney U and Wilcoxon rank sum tests were utilised. A *P*-value less than 0.05 was regarded as indicative of statistical significance.

## Results

### There was no difference In clinical data between the two groups

First, to ensure the reliability of the research results, the clinical baseline data of the two groups of patients were compared. No statistically significant differences were determined in age, gender, pathological type, clinical stage, and other clinical data (*P*>0.05, Table 1), confirming the comparability between the two groups.

**Table 1:** Comparison of clinical baseline data between the two groups of patients.

	Control (n = 107)	Observation (n=82)	χ^2^ (ort) values	*P*-values
Gender			0.586	0.444
male	66 (61.68)	55 (67.07)		
female	41 (38.52)	27 (32.93)		
Age	54.81 ±5.61	55.43±4.93	0.785	0.433
Duration of LC (months)	5.36±1.18	5.59±1.12	1.300	0.195
Pathological type			0.596	0.742
squamous carcinoma	64 (59.81)	45 (54.88)		
adenocarcinoma	34 (31.78)	28 (34.15)		
small cell carcinoma	9 (8.41)	9 (10.98)		
TNM			0.301	0.860
I	26 (24.30)	18 (21.95)		
II	48 (44.86)	40 (48.78)		
III	33 (30.84)	24 (29.27)		
Smoking			0.957	0.328
yes	74 (69.16)	62 (75.61)		
no	33 (30.84)	20 (24.39)		
Drinking			0.545	0.461
yes	34 (31.78)	22 (26.83)		
no	73 (68.22)	60 (73.17)		
Family history of LC			0.560	0.454
yes	3 (2.80)	4 (4.88)		
no	104 (97.20)	78 (95.12)		

### Inflammatory and stress responses were less severe In the observation group

Subsequently, the observation of inflammatory factors and stress response indexes before and after treatment showed no notable inter-group differences in pre-treatment hs-CRF) TNF-α, IL-6, SOD, MDA, and GSH-Px (*P*>0.05). After treatment, the levels of hs-CRF) TNF-α, IL-6, and MDA decreased in both groups; however, the values in the observation group were substantially lower than those in the control group (P<0.05). Conversely, the levels of SOD and GSH-Px increased in both groups, with the values in the observation group being even higher (*P*<0.05, Table 2).

**Table 2:** Comparison of inflammatory factors and oxidative stress in two groups of patients. Note: * indicates P<0.05 compared to before treatment.

Indicators		Control (n = 107)	Observation (n=82)	t values	*P*-values
hs-CRP (mg/L)	before	20.86±6.50	21.57±5.50	0.793	0.429
	after	15.88±5.21*	14.32±4.40*	2.173	0.031
TNF-α (μg/L)	before	25.77±3.04	25.48±3.03	0.636	0.526
	after	22.74±3.39*	20.83±2.68*	4.187	<0.001
IL-6 (ng/L)	before	225.91 ±32.08	218.23±7.27	1.758	0.084
	after	174.51 ±22.98*	166.68±27.65*	2.122	0.035
SOD (U/L)	before	167.10±21.29	163.09±25.72	1.172	0.243
	after	182.62±26.01*	196.02±22.44*	3.727	<0.001
GSH-Px (U/L)	before	83.86±10.99	81.59±11.02	1.404	0.162
	after	90.58±8.29*	95.49±9.82*	3.720	<0.001
MDA (nmol/mL)	before	16.09±3.61	16.79±2.68	1.467	0.144
	after	14.68±2.19*	13.10±3.11*	4.091	<0.001

### Better T-lymphocyte subsets In the observation group

In comparing T lymphocyte subsets, it was likewise observed that no significant differences existed in the test results between the two groups before treatment (*P*>0.05). After treatment, the levels of CD3^+^, CD4^+^, and NK in both groups declined, while the level of CD8^+^ increased (*P*<0.05), confirming the presence of evident immunosuppression among patients. However, through inter-group comparison, it was noted that the post-treatment levels of CD3^+^, CD4^+^, and NK were higher in the observation group versus the control group, and the level of CD8^+^ was lower (*P*<0.05, Table 3).

**Table 3:** Comparison of inflammatory factors and oxidative stress in two groups of patients. Note: * indicates P<0.05 compared to before treatment.

Indicators		Control (n = 107)	Observation (n=82)	t values	P-values
CD3^+^ (%)	before	64.57 ±4.34	63.54±5.30	1.470	0.143
	after	56.78±4.28*	59.77±4.50*	4.668	<0.001
CD4^+^ (%)	before	40.18±4.21	39.63±5.11	0.810	0.419
	after	34.57 ±3.28*	36.82±4.17*	4.174	<0.001
CD8^+^ (%)	before	30.03±4.97	31.13±3.29	1.727	0.086
	after	35.32±3.32*	33.84±3.87*	2.815	0.005
NK (%)	before	22.42±3.87	21.64±3.31	1.465	0.145
	after	16.39±2.48*	18.14±2.03*	5.190	<0.001

### Better gastrointestinal function In the observation group

Statistics demonstrated no difference in G-17, PGI, and PGII between the two groups before treatment (*P*>0.05). After treatment, the levels of G-17, PGI, and PGII in both groups of patients increased; nevertheless, the values were lower in the observation group than in the control group (*P*<0.05, Table 4).

**Table 4:** Comparison of gastrointestinal function between the two groups of patients.

Indicators		Control (n = 107)	Observation (n=82)	t values	P-values
G-17 (pmol/L)	before	9.75±2.33	9.39±2.13	1.073	0.285
	after	14.92±3.55*	12.51 ±2.30*	5.340	<0.001
PGI (ng/ml_)	before	187.99±39.82	189.07±23.35	0.218	0.828
	after	231.34±30.22*	208.89±35.29*	4.704	<0.001
PGII (ng/mL)	before	16.62±4.02	16.33±2.98	0.552	0.582
	after	23.12±3.50*	20.48±3.27*	5.291	<0.001

### Better psychological state and sleep condition In the observation group

**Figure 1:**
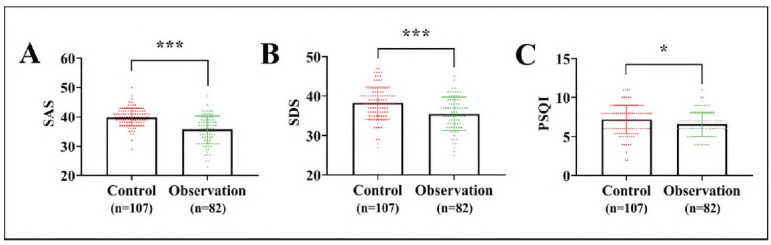
Comparison of psychological state and sleep condition between the two groups of patients showed that the SAS, SDS and PSQI of the observation group were lower than those of the control group.<br>(A) comparison of SAS. (B) comparison of SDS. (C) comparison of PSQI. ***P<0.001, *P<0.05.

Finally, the two groups of patients' psychological state and sleep condition were evaluated. The SAS, SDS, and PSQI scores of the observation group were (35.63±4.70), (35.51 ±4.19), and (6.59±1.56), respectively, all of which were lower compared with the control group (*P*<0.05, Figure 1).

## Discussion

This study found that TCM-characteristic rehabilitation management is beneficial in ameliorating inflammatory and stress responses during postoperative chemotherapy of LC. This substantiates the clinical application value of TCM-characteristic rehabilitation management and provides novel insights for the future treatment of LC.

In TCM, the side effects of chemotherapy are considered to be caused by deficiency of primordial yang, inadequate warming function, internal vacuity of yin-blood, dystrophy of meridians, malnourishment of the meridians, obstruction by wind-cold pathogenic factors, and stagnation of phlegm and blood stasis [18]. The adverse reactions associated with chemotherapy are an established reality, so the optimal approach lies in early prevention and intervention. The key to enhancing patient outcomes lies in incorporating TCM during chemotherapy, thereby attenuating toxicity, augmenting efficacy, and mitigating the adverse sequelae of chemotherapy [19]. TCM-characteristic rehabilitation management is established based on the unique theoretical system and rich clinical experience of TCM. By leveraging medical rehabilitation techniques and rehabilitative treatment modalities, including acupuncture, massage, and TCM therapies, it endeavours to modulate the equilibrium of yin and yang in the human body, promote blood circulation, strengthen immunity, and ultimately attain the purpose of restoring health [20]. The results of this study showed that the inflammation, stress response, and gastrointestinal function in the observation group following treatment were all superior to those in the control group. This implies that TCM-characteristic rehabilitation management can efficaciously enhance the rehabilitation status of LC patients during postoperative chemotherapy, which is also in consonance with the research findings of Xi C et al. [21].

It is widely acknowledged that the toxic and side effects during chemotherapy primarily manifest in two aspects: (1) The abnormal proliferation of cancer cells leads to the excessive consumption of nutrients in the body, resulting in an inadequate energy supply for the proliferation of T lymphocytes and consequent impairment of immune function; (2) The destruction of normal cells by chemotherapy agents gives rise to an intensification of the body's inflammatory response and stress-induced damage, as well as the impairment of the gastrointestinal mucosa [22]. In this process, inflammatory factors such as TNF-α and IL-6 can activate neutrophils and lymphocytes, increase vascular endothelial cells' permeability, regulate other tissues' metabolic activity, and promote the synthesis and release of other cytokines. At the same time, the decrease in the level of antioxidants such as SOD and GSH-Px and the increase in the level of oxidation products such as MDA also indicate the imbalance between oxidation and antioxidant effects in the body, which will also lead to inflammatory infiltration of neutrophils, increased secretion of protease, production of a large number of oxidation intermediates, and play a synergistic damage effect with inflammatory factors. That is, the interaction between the inflammatory response and the stress response largely determines the effectiveness of the patient's chemotherapy and the adverse effects of the process. Therefore, we must focus on LC chemotherapy patients' inflammatory response and stress.

We believe that the positive impacts of TCM-characteristic rehabilitation management on patients are mainly reflected in the following points: (1) TCM acupoint application can continuously stimulate patients, effectively inhibiting macrophages and thereby suppressing the release of inflammatory factors and enhancing the body's immune capacity [23]. (2) Unlike Western medicine, the pharmacological actions of TCM compounds are typically multifaceted. For example, Chen Z et al.'s research indicates that Astragalus is rich in Astragalus polysaccharides, which possess anti-inflammatory and anti-infection effects. Such drugs can also scavenge free radicals, alleviate inflammatory reactions, inhibit cyclooxygenase activity, and play an anti-inflammatory role [24]. Drugs like Radix Aconiti Kusnezoffii preparata invigorate the spleen, strengthen the stomach, regulate qi, and consolidate the exterior. Moreover, acupoint application not only persistently stimulates the acupoints to assist patients In modulating the spleen and stomach function but also effectively sustains the drug efficacy via transdermal absorption, jointly facilitating the restoration of patients' gastrointestinal function [24,25]. (5) More targeted and rational healthy diet guidance Is conducive to ameliorating the malnourished state of patients and Improving their Immune function. What Is more, TCM-characterlstlc rehabilitation management can formulate targeted treatment and nursing plans based on the Individual conditions of patients [26]. This approach better meets patients' needs and actual situations and fully attends to and satisfies patients' physiological requirements, thereby providing a more reliable guarantee for the safety of chemotherapy. G-17 Is an Important hormone secreted by gastric antral cells, stimulating gastric acid secretion and gastrointestinal motility. PGI and PGII are proteins secreted by gastric mucosal cells, and their expression Is an Important Index In determining gastrointestinal function. In this study, the G-17, PGI and PGII of the observation group after treatment were lower than those of the control group, which also confirmed that the gastrointestinal stress reaction of the observation group was lighter, consistent with the above results. In research concerning the treatment of patients with heart failure carried out by Dal M et al. [27], it was unveiled that TCM-characterlstlc management was more conducive to rehabilitating patients' cardiovascular function and preventing complications, which can corroborate our view. Finally, it was noted that TCM-characterlstlc rehabilitation management could efficaciously ameliorate patients' adverse emotional states, such as anxiety and depression and enhance their sleep quality, which Is hypothesised to be due to the use of supplementary nursing Interventions In domains such as environmental care, dietary care, psychological care, and emotional care In TCM-characterlstlc rehabilitation management. These scientific and comprehensive nursing measures effectively ensure the prognostic recovery of patients, ultimately leading to the effective optimisation and Improvement of their emotional states and sleep quality.

However, there are still many limitations In this study that require Improvement. For Instance, there Is a lack of sufficient standardised guidelines to support TCM-characterlstlc rehabilitation management In clinical practice. Besides, the TCM diagnostic and treatment techniques employed In postoperative rehabilitation lack mature operational criteria and diagnostic treatment modalities. Therefore, it is necessary to Investigate further how to Integrate TCM-characterlstlc rehabilitation management with Western medicine treatment and formulate corresponding norms and standards to accelerate Its clinical promotion and popularisation. At the same time, it is necessary to incorporate a greater number of clinical objective indices and conduct extended-term follow-ups with the subjects in this study. This will enable the determination of the prognostic influence of TCM-characteristic rehabilitation management on patients' primordial qi and facilitate the clinical confirmation of the comprehensive impact of TCM-characteristic rehabilitation management on LC patients. Finally, the study population is relatively homogeneous, the results may lack representativeness, and we need to follow up with a large base of cases analysed in conjunction with several hospitals.

## Conclusion

TCM-characteristic rehabilitation management can effectively mitigate inflammatory responses and stress injury in LC patients following postoperative chemotherapy and improve their immune and gastrointestinal functions, providing a more reliable safeguard for patients' prognostic rehabilitation. In the follow-up, continuous refinement of the clinical standardised utilisation of TCM-characteristic rehabilitation management is warranted to ensure more efficacious promotion and popularisation.

## Dodatak

### Author contributions

XN and YYT conceived and designed the project and wrote the paper. PS generated and analysed the data. XNZ and YYT made equal contributions to this work as co-first authors. All authors gave final approval of the version to be published and agreed to be accountable for all aspects of the work.

### Availability of data and materials

The data supporting this study's findings are available from the corresponding author upon reasonable request.

### Funding

This study was supported by the Scientific Research Project of Chinese Medicine in Heilongjiang (ZHY2025-054).

### Acknowledgements

Not applicable.

### Conflict of interest statement

All the authors declare that they have no conflict of interest in this work.
